# Treatment outcomes in pediatric intestinal failure patients with ambulatory *Candida* central line‐associated bloodstream infections with and without central venous line removal: A retrospective case series

**DOI:** 10.1002/ncp.70000

**Published:** 2025-07-27

**Authors:** Hamza Hassan Khan, Candi S. Jump, Jessica Bauer, Qian Wu, Stephen Thacker, Allison Ross Eckard

**Affiliations:** ^1^ Department of Pediatrics, Division of Gastroenterology Medical University of South Carolina Charleston South Carolina USA; ^2^ Department of Pediatrics, Division of Gastroenterology University of Arkansas for Medical Sciences Little Rock Arkansas USA; ^3^ Department of Pediatrics Medical University of South Carolina Charleston South Carolina USA; ^4^ Department of Population and Quantitative Health Sciences Case Western Reserve University Cleveland Ohio USA; ^5^ Department of Pediatrics, Division of Infectious Diseases Medical University of South Carolina Charleston South Carolina USA; ^6^ Department of Medicine, Division of Infectious Diseases Medical University of South Carolina Charleston South Carolina USA

**Keywords:** central line‐associated bloodstream infections, central venous line, *Candidemia*, intestinal failure, intestinal transplantation, line salvage, pediatrics

## Abstract

**Background:**

Lack of central venous line (CVL) sites is a common indication for intestinal transplantation in intestinal failure (IF) patients. For treatment of central line‐associated bloodstream infections (CLABSIs), many pediatric gastroenterologists preserve CVL access, but line removal is typically recommended for *Candida* sp CLABSI due to high risk of systemic complications. However, no data exist on outcomes for IF patients treated for *Candida* sp CLABSI. This study aims to assess if CVL preservation increases the risk of complications or recurrence.

**Materials and Methods:**

A retrospective chart review was conducted for children <18 years of age with IF and *Candida* sp CLABSI between 2012 and 2023. Patients with blood cultures positive for *Candida* sp from the CVL were included. Each CLABSI event was analyzed for key variables.

**Results:**

Twelve patients were included, with 18 events (median age 6.4 years). *Candida* species identified included *C albicans* (33.3%) and *C parapsilosis* (38.9%). Antifungal therapy exceeded 14 days in all events, and ethanol lock therapy (70% concentration) was used in 72%. CVL was removed in 44.4% of events. Five patients had multiple events, with *Candida* species identified in subsequent infections. No secondary site seeding or long‐term sequelae occurred, and no patients died.

**Conclusion:**

Our data suggest that CVL preservation in pediatric IF patients with *Candida* sp CLABSI is feasible without increased complications or mortality. Limitations include small sample size and retrospective design.

## INTRODUCTION

Pediatric intestinal failure (IF) patients require long‐term central venous access to obtain life‐sustaining parenteral nutrition support. Loss of central access sites is an increasingly common reason for intestinal transplantation.^1^ Thus, attempted central venous line (CVL) salvage for central line‐associated bloodstream infections (CLABSIs) is standard practice for intestinal rehabilitation programs as conveyed in a position paper by the North American Society of Pediatric Gastroenterology, Hepatology, and Nutrition. Notably, however, there are no specific recommendations for *Candida* sp CLABSIs.^2^

This contrasts with treatment practices of many pediatric infectious disease physicians in which *Candida* CLABSI management often includes routine CVL removal owing to *Candida*'s propensity to develop biofilms, making organism eradication difficult, and its tendency to seed hematogenously to distant sites, potentially causing serious morbidity and mortality. In fact, the Infectious Diseases Society of America explicitly recommends CVL removal for *Candida* CLABSIs.^3^

Opposing recommendations highlight the need to investigate *Candida* CLABSI management in pediatric patients with IF because the risk‐benefit ratio differs from other patient populations and may support increased attempts at CVL salvage. To date, there are no published studies specifically evaluating treatment outcomes with retained vs removed CVLs for *Candida* CLABSIs in this unique population. Thus, this study was conducted to examine whether CVL retention was associated with infection recurrence or serious sequelae. Data from our retrospective review are intended to inform future trials and determine the feasibility of developing standardized treatment pathways that maximize eradication rates while preserving CVL access.

## METHODS

### Study design and cohort assembly

We conducted a retrospective cohort study in children <18 years old treated for IF, as previously defined,^4^ at our institution between January 1, 2012, and June 30, 2023. Patients were identified via our pediatric gastroenterology division's IF cohort database and assessed for eligibility by review of the electronic medical records (EMR). IF patients were included if they met the a priori definition of *Candida* CLABSI: hospital admission from the ambulatory setting with clinical signs and/or symptoms suggestive of CVL infection and ≥1 blood culture positive for *Candida* species obtained from the CVL. All organisms belonging to the genus *Candida* at the start of our investigation were included in the analysis; since then, mycologists have updated nomenclature for several fungi known to cause CLABSIs and previously designated as *Candida* species. Each admission was considered a discrete event. Hospital‐acquired CLABSIs were exclusionary. Exclusion criteria included patients >18 years of age at the time of the *Candida* CLABSI event, hospital‐acquired CLABSIs, and pediatric IF patients without *Candida* CLABSI.

### Data collection

Patient‐level EMR data were collected, including demographics, intestinal anatomy, parenteral nutrition support, home CVL care regimens, and prior CVLs and CLABSIs. Presenting clinical findings, concomitant non‐*Candida* infections, microbiological data, antimicrobial treatment, hospital course, and CVL retention vs removal were recorded for each event. Escalation of care was defined as the need to transfer the patient from the acute care unit to the intensive care unit for hemodynamic instability related to the *Candida* CLABSI.

### Outcomes and classifications

Primary outcomes included measures of clinical disease and treatment outcomes (ie, severity of illness, development of secondary sites of infection, serious sequelae, and infection recurrence) in patients with retained vs removed CVLs for each CLABSI event. For patients with >1 event, subsequent events were assessed for infection recurrence if the patient was found to have CLABSI with the same *Candida* species ≤30 days from completion of antifungal therapy for the prior event.

### Statistical analyses

Data were first summarized by patient and then by event. Event data were described by median (and interquartile range) for continuous measures and frequency (and percent) for nominal variables. Between‐group comparisons (ie, CVL retained vs removed) were conducted using two‐sample *t* tests or Wilcoxon rank sum tests, as appropriate. The Benjamini‐Hochberg procedure was used to adjust for multiple testing given the small sample size relative to the number of variables analyzed. Because of the study's exploratory nature, we selected a false discovery rate of 0.20 for correction of *P* values. Descriptive statistics were calculated using Microsoft Excel. Other analyzes were performed using Stata 18.0 and R 4.3.0 statistical software. The project was certified by the institution's institutional review board as quality improvement.

## RESULTS

Thirty‐five pediatric patients with IF were identified. Among these, 12 patients met inclusion criteria. Collectively, these 12 patients had a total of 18 *Candida* CLABSI events. Patient characteristics and event details are summarized in Table 1. None of the excluded patients had a *Candida* CLABSI history, and there were no inpatient *Candidemia* events in IF population at our center.

**Table 1:** Patient characteristics and summary of *Candida* CLABSI events.

Patient no.	Event no. for patient	Days between event presentations	Age, years	Empiric antifungal treatment^a^	*Candida* species^b^	Days of positive *Candida* blood cultures	Concomitant non‐*Candida* fungemia and/or bacteremia (additional infection[s] present)	Days of positive non‐*Candida* blood cxs	Hemodynamic instability^c^	ELT at the time of the event	CVL retained	Days after presentation CVL was removed
Patients with 1 event												
Patient 1	Event 1	N/A	2.5	No	*C albicans*	4	*Lactobacillus* sp	2	No	Yes	Yes	N/A
Patient 2	Event 1	N/A	1.4	No	*C parapsilosis*	—	No	N/A	Yes	—	Yes	N/A
Patient 3	Event 1	N/A	0.1	No	*C parapsilosis*	4	CoNS	2	No	Yes	Yes	N/A
Patient 4	Event 1	N/A	15.0	No	*C parapsilosis*; *C tropicalis*	5	*Bacillus* sp (*P aeruginosa*; *P mirabilis* pyelonephritis)	1	No	No	No	4
Patient 5	Event 1	N/A	6.5	No	*C tropicalis*	2	No	N/A	Yes	Yes	No	1
Patient 6	Event 1	N/A	1.4	No	*C parapsilosis*	2	CoNS	—	No	No	No	8
Patient 7	Event 1	N/A	12.8	No	*C albicans*	—	*S hemolyticus*	—	No	No	No	2
Patients with >1 event^d^												
Patient 8	Event 1	N/A	6.2	No	*C glabrata*	1	No (SARS‐CoV‐2 URI)	N/A	No	Yes	Yes	N/A
	Event 2	224	6.8	Yes	*C glabrata*	2	No	N/A	No	Yes	Yes	N/A
Patient 9	Event 1	N/A	16.9	No	*C albicans*	5	CoNS, *Lactobacillus* sp	1	No	Yes	Yes	N/A
	Event 2	336	17.8	No	*C albicans*	10	CoNS, *Enterococcus hirae (patellar osteomyelitis)*	4	No	Yes	Yes	N/A
Patient 10	Event 1	N/A	6.1	No	*C albicans*	1	No	N/A	No	Yes	No	7
	Event 2	3558	15.8	No	*C albicans*	1	*P aeruginosa*	2	No	Yes	Yes	N/A
Patient 11	Event 1	N/A	12.3	No	*C parapsilosis*	3	*E cloacae*; *K pneumoniae*; *Streptococcus* sp	1	No	Yes	No	2
	Event 2	179	12.8	No	*C parapsilosis*; *C lusitaniae*	2	*E cloacae*; *K pneumoniae*; *E faecalis*; *C freundii*; *Enterococcus* species; *K ohmeri*	3	Yes	Yes	Yes	N/A
Patient 12	Event 1	N/A	0.4	No	*Candida* sp^e^	2	*A ursingii*	1	No	No	Yes	N/A
	Event 2*	34	0.5	Yes	*C lusitaniae*	1	*Acinetobacter* sp (rhino/enterovirus URI)	1	No	Yes	No	2
	Event 3	36	0.6	Yes	*C parapsilosis*	1	*E faecium*; *K pneumoniae*; *Pseudomonas* sp (not *aeruginosa*)	2	No	Yes	No	11

*Note*: All patients with non‐Hispanic ethnicity except patient No. 3. Dashed line represents missing data because of retrospective nature of the study.

Abbreviations: CLABSI, central line‐associated bloodstream infection; CoNS, coagulase negative *Staphylococcus*; CVL, central venous line; ETL, ethanol lock therapy; URI, upper respiratory infection.

^a^ Intravenous antifungal therapy started at initial presentation prior to positive blood culture.

^b^ Mycologists have recently updated nomenclature for *C glabrata* and *C lusitaniae* to *Nakaseomyces glabrata* and *Clavispora lusitaniae*, respectively.

^c^ Hemodynamic instability during admission that required escalation of care due to *Candida* CLABSI.

^d^ All subsequent events occurred ≥6 months from prior except in patient 12 where events 2 and 3 occurred 18 and 21 days from completion of antifungal therapy for the prior event, respectively.

^e^ Reported as “not *C albicans* or *C glabrata*” but isolate was not speciated further.

* Designated as “possible recurrence”because of event ≤30 days from end of treatment for prior CLABSI, but incomplete isolate speciation in Event 1 prevented confirmation of same *Candida* species in Event 2.

Underlying IF etiologies included the following: necrotizing enterocolitis (*n* = 5), gastroschisis (*n* = 2), small bowel atresia (*n* = 1), small bowel atresia plus volvulus (*n* = 1), total intestinal aganglionosis (*n* = 1), motility disorder (*n* = 1), and strangulated internal hernia (*n* = 1). All patients had tunneled external catheters with all but one composed of silicone (1 = polyurethane). The tunneled external catheters were single lumen in all events except patient 10 (event 1 and event 2) in which it was double lumen. All patients were receiving intravenous fat emulsions. Ethanol lock therapy (70% concentration) was used in 72%, and no antifungal locks were used in any patients. All patients presented with fever; no patient presented with other clinical signs of sepsis, including hemodynamic instability requiring escalation of care, related to their CLABSIs. All patients had central and peripheral blood culture, and intravenous antibacterial therapy was started for all events at presentation. A total of 6 of 18 (33.3%) events occurred among patients with prior *Candida* CLABSIs with median of 201.5 days between event presentations; five of them met the *priori* definition of separate events. Days between these event presentations were 224 days (patient 8, event 2), 336 days (patient 9, event 2), 3558 days (patient 10, event 2), 179 days (patient 11, event 2), and 36 days (patient 12, event 3). We observed one possible recurrence (patient 12, event 2). This event occurred 18 days from completion of treatment (intravenous antifungal therapy for 14 days from negative blood culture) for the preceding event in which the CVL was retained; however, complete organism speciation was not performed. The CVL was replaced during this second event. The patient (patient 12, event 3) was admitted a third time for another CLABSI with a different *Candida* species 21 days from completion of treatment for the second event. Of all of these events, intravenous antifungal therapy was started at presentation in three (50%) events and after blood cultures detected yeast in the other three (50%) events. In all events but one (patient 8, event 2), the *Candida* isolate was susceptible to the initial antifungal used (determined based on minimum inhibitory concentration, clinical improvement, and/or blood sterilization).

Table 2 depicts patient characteristics among events with retained vs removed CVLs. There were no statistically significant differences between groups for any measured variable. No event was associated with suspected or confirmed primary site of infection (other than the CVL) or secondary sites of infection, severe infection, or serious sequelae. In the two events requiring escalation of care in the CVL retained group, both patients developed hypotension; patient 2 required several hours of inotropic support, and patient 11 (event 2) responded to intravenous fluid boluses. Patient 11 (event 2) also had concomitant bacteremia, improved quickly after additional treatment, and both patients were transferred back to the acute care unit in <2 days.

**Table 2:** Patient characteristics for events with retained vs removed CVLs.

Characteristic at time of event	CVL retained (*N* = 10)	CVL removed (*N* = 8)	*P* value
Demographics			
Age, years, median (interquartile range)	6.5 (1.4−15.8)	6.3 (1.0−12.6)	0.66
Sex, male, *n* (%)	8 (80.0)	7 (87.5)	0.67
Race, Black, *n* (%)	5 (50.0)	3 (37.5)	0.60
Clinical history			
Percentage of total calories obtained parenterally, median (interquartile range)	62.5 (43.0−100)	51.5 (43.5−70.5)	0.42
Total lifetime number of CVLs, median (interquartile range)	2 (2−6)^a^	2 (1−3)^a^	0.23
Total number of CVL days, median (interquartile range)	588.50 (86.00–2196.00)^b^	133.00 (120.00–146.00)^c^	0.50
*Candida* CLABSI rate (per 1000 CVL days)	1.02	7.52	
CVL replacement rate (per 1000 CVL days)	1.44	11.3	
Relevant treatment and confounding factors, *n* (%)			
Antifungal therapy started at initial presentation	1 (10.0)^a^	2 (25.0)	0.40
Concomitant non‐*Candida* fungemia or bacteremia	7 (70.0)	6 (75.0)	0.81
Ethanol lock therapy utilized (dwell time range: 2–7 h/day)	8 (88.9)^a^	5 (62.5)	0.20
Hospital and treatment course			
Hemodynamic instability during admission requiring escalation of care due to *Candida* CLABSI, *n* (%)	2 (20.0%)	1 (12.5%)	0.67
Duration of antifungal therapy, days	16 (15−17)^a^	14 (14−17)^a^	0.30
Microbiological outcomes			
Duration of positive *Candida* blood cultures, days, median (interquartile range)^d^	2 (2−4)^a^	2 (1−3)^a^	0.33
Positive *Candida* peripheral venous blood culture(s), *n* (%)	6 (66.7%)^a^	4 (57.1%)^a^	0.70
Time to *Candida* blood sterilization from start of antifungal therapy, h, median (interquartile range)	48 (36−95)	36 (24−84)	0.44
Duration of positive non‐*Candida* fungal or bacterial cultures, days, median (interquartile range)	2 (1−3)	1 (1−2)^e^	0.13

*Note*: No characteristic was statistically significant after applying Benjamini‐Hochberg procedure using a false discovery rate of 0.20.

Abbreviations:CLABSI, central line‐associated bloodstream infection; CVL, central venous line.

^a^ Data missing for one patient.

^b^ Data missing for four patients.

^c^ Data missing for six patients.

^d^ Includes cultures obtained from any intravascular line or peripheral blood draw.

^e^ Data missing for two patients.

## DISCUSSION

To our knowledge, this is the first study specifically investigating treatment outcomes in retained vs removed CVLs among pediatric patients with IF with ambulatory *Candida* CLABSIs. We observed no discernible differences in complication rates between groups. No event was associated with suspected or confirmed secondary sites of infection, severe infection, or serious sequelae; most patients had abdominal ultrasound and echocardiogram performed, however the process of screening for secondary sites of infection was not uniform in all of the patients. Of the six patients with prior *Candida* CLABSIs, there was one possible recurrence in a patient with an event that occurred soon after completion of antifungal therapy for a previous *Candida* CLABSI with retained CVL. Because the initial *Candida* isolate was not fully speciated, however, we designated this event as a possible rather than likely recurrence. Importantly, this patient was treated for a third *Candida* CLABSI with a different species shortly after the second event with CVL removal, indicating this patient's CVL salvage failure may have been due to patient‐specific CLABSI risk factors rather than because of the causative organism. Regardless, this patient did not suffer serious harm from CVL retention. Interestingly, although there was no statistically significant difference between the utilization of ethanol lock therapy (70% concentration) between the CVL retained and removed groups, the time between subsequent *Candida* CLABSI events was noted to be ≥6 months in the group with ELT (with the exception of patient 12). In addition, although the *Candida* CLABSI and CVL replacement rates were high in the CVL removed group compared with that of the CVL retained group, no definitive conclusion could be made given the missing data in both groups because of the retrospective nature of the study.

Pediatric IF patients are at high risk of CLABSIs because of impaired gut integrity that promotes microbial translocation compounded by long‐term central venous access and additional host and treatment factors.^4^, ^5^, ^6^, ^7^ However, the incidence of *Candida* CLABSI in this population is poorly defined. We observed relatively low rates in our investigation that included over 10 years of data comparable to other studies.^7^, ^8^, ^9^ In fact, 66% of our cohort did not experience a *Candida* CLABSI. This contrasts with much higher bacterial CLABSI rates, as corroborated by our data in which 72% of events had concomitant bacteremia.

Polymicrobial infections complicated the interpretation of treatment outcomes and management decisions. However, concomitant bacteremia, like other investigated factors, did not delineate retained vs removed CVLs. Medical decision‐making appeared random, likely related to individual providers' practice differences and/or comfort levels. This highlights the need for pediatric intestinal failure (IF)‐specific studies to determine best practices for this unique population rather than lumping them with patients such as those with malignancies or hospital‐acquired CLABSIs whose clinical and microbiological characteristics and risk‐benefit analyzes differ dramatically.^10^

In fact, recent studies show CVL salvage attempts are reasonable among stable pediatric IF patients with bacterial CLABSIs, even with controversial bacteria like *Staphylococcus aureus*.^7^, ^11^ Small numbers of fungal CLABSIs were included in one pediatric IF study that evaluated retained vs removed CVLs for all types of CLABSIs.^12^ This study provided critical data regarding CVL salvage in this population. We contend, however, that ambulatory *Candida* CLABSIs have distinctive clinical and microbiological properties and deserve independent scrutiny.

Our study suggests CVL salvage is a reasonable treatment approach in pediatric patients with IF with ambulatory *Candida* CLABSIs. The data are limited by small sample size, lack of molecular testing in certain cases, and a retrospective study design, which temper our conclusions but nevertheless lay the groundwork for future studies. Prospective, multicentered trials are warranted to facilitate the development of standardized national guidelines specific to these unique, high‐risk patients with *Candida* CLABSIs that bridge the clinical practice gap between pediatric gastroenterology and infectious diseases subspecialists.

## AUTHOR CONTRIBUTIONS

Hamza Hassan Khan, Candi S. Jump, Jessica Bauer, Qian Wu, Stephen Thacker, and Allison Ross Eckard substantially contributed to the conception or design of the work; the acquisition, analysis, or interpretation of data for the work; the drafting of the work or revising it critically for important intellectual content; the final approval of the version to be published; and agreement to be accountable for all aspects of the work in ensuring that questions related to the accuracy or integrity of any part of the work are appropriately investigated and resolved.

## CONFLICT OF INTEREST STATEMENT

None declared.
